# Estimating Transmission Parameters for COVID-19 Clusters by Using Symptom Onset Data, Singapore, January–April 2020

**DOI:** 10.3201/eid2702.203018

**Published:** 2021-02

**Authors:** Sheryl Hui-Xian Ng, Palvinder Kaur, Cécile Kremer, Woan Shin Tan, Aidan Lyanzhiang Tan, Niel Hens, Matthias Paul Toh, Kiok Liang Teow, Palvannan Kannapiran

**Affiliations:** National Healthcare Group, Singapore (S.H.-X. Ng, P. Kaur, W.S. Tan, A.L. Tan, K.L. Teow, P. Kannapiran);; Hasselt University, Hasselt, Belgium (C. Kremer, N. Hens);; University of Antwerp, Antwerp, Belgium (N. Hens);; National Centre for Infectious Diseases, Singapore (M.P. Toh);; National University of Singapore, Singapore (M.P. Toh)

**Keywords:** COVID-19, coronavirus disease, SARS-CoV-2, severe acute respiratory syndrome coronavirus 2, viruses, respiratory infections, zoonoses, Singapore

## Abstract

We estimated the generation interval distribution for coronavirus disease on the basis of serial intervals of observed infector–infectee pairs from established clusters in Singapore. The short mean generation interval and consequent high prevalence of presymptomatic transmission requires public health control measures to be responsive to these characteristics of the epidemic.

A systematic review estimated that the basic reproduction number (R_0_) for coronavirus disease (COVID-19) is 2–3 (*1*). However, R_0_ alone is insufficient to characterize an epidemic. The distribution of the serial interval (i.e., the length of time between symptom onset of 2 cases) has been estimated for COVID-19; mean intervals range from 3.1 to 7.5 days (*2*,*3*). Estimation of the generation interval (*T*_g_) (i.e., the length of time between the points of infection for 2 linked cases) is less common. Although studies have reported means of 3.3 and 5.0 days (*4*; Li et al., unpub. data, ), Ganyani et al. (*5*) estimated the mean (+ SD) of *T*_g_ to be 3.9 (+ 2.7) days, on the basis of which they estimated that 66% (95% credible interval [CrI] 45%–84%) of transmission occurred before symptoms. Another study of 77 pairs estimated the same proportion to be 44% (95% CI 25%–69%) (*6*). Because conventional outbreak control measures are centered around isolation, contact tracing, and treatment of symptomatic case-patients, a high prevalence of presymptomatic transmission (*p*) would warrant shifting measures to address potential transmission among persons with no apparent symptoms (*7*). Hence, to inform control measures for the outbreak in Singapore, we generated estimates of *T*_g_, R_0_, and *p* by using published symptom onset data for COVID-19 cases in Singapore.

## The Study

We implemented a cross-sectional study design to estimate *T*_g_, R_0_, and *p* for the COVID-19 outbreak in Singapore during January 23–April 6, 2020. Given that containment measures were initiated over the duration of the study, we considered R_0_ 𝑡o be the effective reproduction number of the outbreak. All confirmed COVID-19 cases classified by the Ministry of Health of Singapore (MOH) as linked to a local cluster were included in this analysis. Information on case number, cluster, patient age and sex, imported status, date of symptom onset (DOO), and known contacts who have also been confirmed as case-patients were extracted from daily press releases published by MOH. DOOs for cases that were not available from press releases were extracted from a similar anonymized dataset of COVID-19 admissions to the National Centre for Infectious Diseases, Singapore. Cases with DOOs not available from that dataset were subsequently excluded from analysis. Our study was approved by the ethics review board of National Healthcare Group, Singapore.

We identified index cases and potential infectors of each case-patient on the basis of available information of the case-patients’ known contacts, published case links, and a heuristic to sensibly include potential infectors who could have transmitted the infection to the case-patients (Appendix). We subsequently used the infector–infectee pairs constructed to estimate the serial and generation interval distribution.

Assuming the same incubation period with mean (+ SD) of 5.2 (+ 2.8) days, we replicated the Bayesian Markov chain Monte Carlo procedure detailed in Ganyani et al. (*5*) to estimate the mean (SD) of the *T*_g_ (Appendix). With the estimated parameters, we constructed the distribution of R_0_ and subsequently *p* by simulating infections and computing the proportion of presymptomatic transmissions. We conducted subgroup analyses for case-patients with multiple, family, or no contacts, and for clusters with no missing DOO. We conducted sensitivity analyses estimating the distribution of R_0_ by using resampled values from a 95% CI of the epidemic growth rate and group-specific rates. For each distribution, we reported the median and 95% CrI. All analyses were conducted by using RStudio 1.2.5033 (https://rstudio.com).

**Figure 1:**
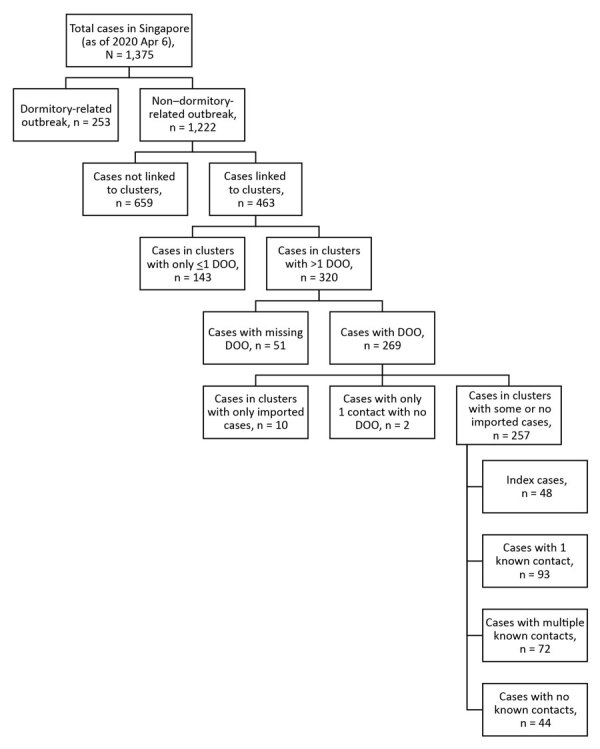
Inclusion and exclusion of coronavirus disease case-patients for analysis, Singapore, January–April 2020. DOO, date of symptom onset.

**Figure 2:**
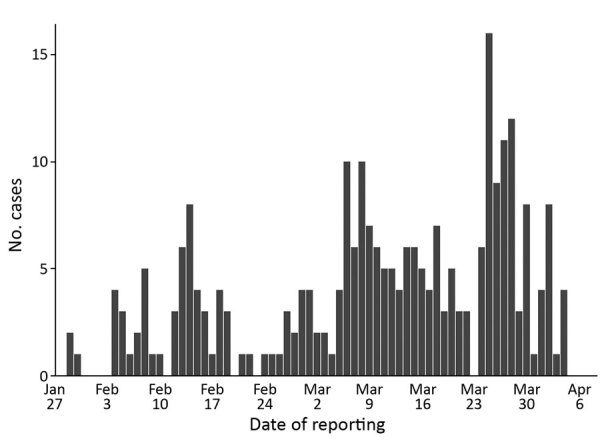
Epidemic curve of coronavirus disease clusters, Singapore, January–April 2020.

A total of 1,375 confirmed cases had been reported as of April 6, 2020, and we applied our exclusion criteria to obtain a final sample size of 257 cases (Figure 1). We have summarized sample characteristics (Table 1) and the spread of cases over time (Figure 2). Because 48 index case-patients had no known infector, a maximum of 209 infector–infectee pairs were constructed for analysis.

**Table 1:** Characteristics of coronavirus disease case-patients in study estimating transmission parameters for coronavirus disease clusters by using symptom onset data, Singapore, January–April 2020

Characteristic	No. (%)*
Age, y, median (25th–75th percentile)	47 (30–59)
Sex	
M	121 (47.1)
F	136 (52.9)
Imported	24 (9.3)
Cluster size, N = 51	
2 cases	28 (54.9)
3 cases	12 (23.5)
>4 cases and above	11 (21.6)

*Values are no. (%) unless otherwise indicated.

Analyzing the 209 pairs, we estimated the mean *T*_g_ to be 3.44 (95% CrI 2.79–4.11) days, with an SD of 2.39 (95% CrI 1.27–3.45) days (Table 2). This estimate corresponded to an R_0_ of 1.09 (95% CrI 1.08–1.11) and *p* of 0.72 (95% CrI 0.64–0.80). We estimated the serial interval distribution (Appendix Table 1) and convergence plots for all analyses (Appendix Figure 3).

**Table 2:** Estimates of transmission parameters of coronavirus disease clusters, Singapore, January–April 2020 *

Infectee type	Median (95% credible interval)			
	Mean	SD		*p*
All case-patients, N = 209	3.44 (2.79–4.11)	2.39 (1.27–3.45)	1.09 (1.08–1.11)	0.72 (0.64–0.80)
Case-patients with only 1 known contact, n = 93	3.93 (3.00–4.93)	2.63 (1.10–4.31)	1.11 (1.08–1.14)	0.65 (0.54–0.76)
Case-patients with only multiple or no known contacts, n = 116	3.03 (2.13–3.97)	2.45 (0.86–4.21)	1.08 (1.06–1.11)	0.76 (0.65–0.86)

*p, presymptomatic proportion; R_0_, basic reproduction number; *T*_g_, generation time.

Examining the 93 pairs with only 1 known contact, the estimates for mean *T*_g_, SD *T*_g_, and R_0_ increased, whereas *p* decreased (Table 2). The 116 pairs that required identification of potential infectors had a shorter mean *T*_g_ and a higher *p* in comparison (Table 2). Subgroup analyses are summarized in Appendix Table 2. However, the chains for pairs with family or no known contact exhibited poor convergence, and estimates were not reported. Sensitivity analyses using resampled growth rates and group-specific rates did not yield estimates differing from those of the main analyses (Appendix Table 3).

## Conclusions

The mean generation interval of the COVID-19 outbreak in Singapore was estimated at 3.44 days, suggesting that an infected person would be expected to pass on an infection to another person in 3 days, within the range of 3.3–5.0 days reported by other studies (*4**,**5*; Li et al., unpub. data). Pairs with only 1 known contact yielded a larger estimate of 3.93 days, whereas pairs for whom infectors were identified had a shorter mean generation interval of 3.03 days. These results suggest that we might best report the upper bound of estimates, accounting for the presence of unclear transmission links within the clusters.

The estimated was slightly >1, higher than other estimates reported as of March 31, 2020 (*8*). We observed a high *p*, potentially a result of prompt isolation of symptomatic case-patients (M. Casey et al., unpub. data, https://doi.org/10.1101/2020.05.08.20094870). This higher proportion might also be attributable to our allowance of infector DOOs to be up to 3 days after their infectees’ DOOs, establishing the plausibility of presymptomatic transmission. We acknowledge that this cutoff would have an influence on our eventual estimates. Although negative serial intervals >3 days have occurred in other studies (*5*; Z. Du et al., unpub. data, ), we chose a conservative cutoff of 3 days consistent with He et al. (6), where 9% of transmissions would occur before 3 days before DOO.

Nonetheless, the high prevalence of presymptomatic transmission in the community requires public health strategies to be responsive to this characteristic to remain effective. Universal wearing of masks in the community might reduce the likelihood of transmission through saliva and respiratory droplets (*9*). In place of testing when symptoms are observed, universal testing of persons living in or working with confined populations should be prioritized to mitigate the risk for transmission of the infection into these populations (*10*). Contact tracing should be modified to include the period before symptom onset (*6**,**7*) and should adopt a digital approach to be more comprehensive and less labor intensive (*4*).

Our study generated estimates that accounted for the uncertainty arising from multiple potential infectors and a small sample size, which contributes to the scarce information about disease characteristics. Because we dropped cases without a reported DOO, and DOO data and contact information were self-reported, our estimates might be subject to selection, self-report, and recall biases. Our estimation approach assumed equal probability of infecting among potential infectors, although a higher likelihood of transmission among household contacts has been suggested (*11*). We also did not account for the potential formation of cyclical infector networks, although their effects on the estimates have been demonstrated to be limited (*12*). Nevertheless, our estimates contribute to knowledge about the transmission dynamics of COVID-19 and have implications for control measures.

Appendix Additional information about estimating transmission parameters for COVID-19 clusters by using symptom onset data, Singapore, January–April 2020.
