# Supplementary material for: Estimating Transmission Parameters for COVID-19 Clusters by Using Symptom Onset Data, Singapore, January–April 2020
Source: Emerg Infect Dis. 2021 Feb;27(2):582–5. doi: 10.3201/eid2702.203018 (PMC7853533; doi:10.3201/eid2702.203018)
Supplement: Appendix — Additional information about estimating transmission parameters for COVID-19 clusters by using symptom onset data, Singapore, January–April 2020. [file 20-3018-Techapp-s1.pdf]

Article DOI: https://doi.org/10.3201/eid2702.203018

Estimating Transmission Parameters for COVID-19 Clusters by Using Symptom Onset Data, Singapore, January–April 2020

Appendix

Identifying index cases within each cluster

Within each cluster, an index case was either

(i) a primary case determined by epidemiologic investigations by the Ministry of Health, Singapore,

(ii) an imported case,

(iii) a case with the earlier date of symptom onset (DOO) in a cluster with only one other case,

(iv) a case with the earliest DOO in the cluster, and no subsequent cases with a DOO within 3 days after it.

The index cases would not have any possible infectors by definition. Clusters with no cases satisfying the criteria (i) to (iv) would not have a defined index case.

Heuristic to identify potential infectors of cases

We identified potential infectors of each case based on available information of the cases' known contacts, published case links (*1*,*2*), and a heuristic to sensibly include a pool of candidates who could have transmitted the infection to the cases.

We defined a potential infector as an infector with a DOO within a period spanning 14 days before and 3 days after the DOO of the case. These thresholds were chosen to ensure that an infector-infectee pair would generate a plausible serial interval. We described two scenarios in Appendix Figure 1 to illustrate the implications of the thresholds. The first scenario would occur when the DOO of the infectee is after that of the infector (Appendix

Figure 1). As the maximum incubation period of COVID-19 has been suggested to be 14 days (*3*), we have considered the maximum plausible serial interval to be 14 days. The second scenario would occur when the DOO of the infectee is before that of the infector (Appendix Figure 1). As analyses by He *et al* found only 9% of transmission to occur 3 days before an infector's DOO and recommended for contact tracing to include this window (*4*), the minimum serial interval we would consider would be −3 days.

We then constructed infector-infectee pairs of cases by assigning an infector to each case. Within clusters that have only two cases, the index case was assigned as the infector for the other case in the cluster. Within larger clusters, cases that have only one known contact were assigned this contact as their infector (Appendix Figure 2).

For cases with multiple contacts, a potential infector was randomly selected from the pool of contacts using an independence sampler in the Markov Chain Monte Carlo (MCMC) algorithm. For cases with no known contacts, a potential infector was similarly selected from other cases within the same cluster. If a case had no potential infectors with a DOO within the period for plausible serial intervals, the case's known contacts were nonetheless assigned as potential infectors.

Estimating the distributions of transmission parameters

Assuming the same incubation period with mean of 5.2 days and standard deviation of 2.8 days (*5*), we replicated the Bayesian MCMC procedure as detailed in Ganyani *et al* (*6*), to estimate the mean and standard deviation of the generation interval (T_g) distribution. Briefly, the infectors assigned and parameters of the T_g distribution were updated in a two-step process. The unknown infector-infectee links were updated using an independence sampler. Uniform priors were assigned to the parameters of the generation interval distribution and updated using a random-walk Metropolis-Hastings algorithm with a uniform proposal distribution (*7*). The posterior distribution was modeled using 3,000,000 iterations of which the first 500,000 were discarded as burn-in, thinning applied every 200 iterations, resulting in 12,500 iterations of accepted parameter estimates.

With the 12,500 instances of estimates and the spread of cases, we computed the corresponding effective R_0 estimates using the relationship $R_0 = e^{r\mu-(\frac{1}{2}r^2\sigma^2)}$, where *r* is the growth rate of the epidemic and μ, σ are the mean and standard deviation of the generation interval distribution (*8*). Using the same parameter estimates and the incubation period

parameters assumed above, we simulated 1000 transmissions per iteration and computed the pre-symptomatic proportion as the proportion of transmissions that occurred before the end of the infector's incubation period. For each distribution, the median and 95% credible interval were reported.

References

1. Abu Baker J, Yong M. Chinese New Year gathering identified as missing link between COVID-19 church clusters. CNA [cited 2020 Jun 12]. https://www.channelnewsasia.com/news/singapore/covid19-coronavirus-missing-link-found-church-clusters-12469236

2. Kurohi R, Chee K, Goh T, Khalik S. 9 in 10 coronavirus patients in Singapore are linked to worker dormitories. The Straits Times [cited 2020 Jun 12]. https://www.straitstimes.com/singapore/health/novel-coronavirus-cases-in-singapore

3. Lauer SA, Grantz KH, Bi Q, Jones FK, Zheng Q, Meredith HR, et al. The incubation period of coronavirus disease 2019 (COVID-19) from publicly reported confirmed cases: estimation and application. Ann Intern Med. 2020;172:577–82. PubMed https://doi.org/10.7326/M20-0504

4. He X, Lau EHY, Wu P, Deng X, Wang J, Hao X, et al. Author correction: temporal dynamics in viral shedding and transmissibility of COVID-19. Nat Med. 2020;26:1491–3. PubMed https://doi.org/10.1038/s41591-020-1016-z

5. Zhang J, Litvinova M, Wang W, Wang Y, Deng X, Chen X, et al. Evolving epidemiology and transmission dynamics of coronavirus disease 2019 outside Hubei Province, China: a descriptive and modelling study. Lancet Infect Dis. 2020;20:793–802. PubMed https://doi.org/10.1016/S1473-3099(20)30230-9

6. Ganyani T, Kremer C, Chen D, Torneri A, Faes C, Wallinga J, et al. Estimating the generation interval for coronavirus disease (COVID-19) based on symptom onset data, March 2020. Euro Surveill. 2020;25:2000257. PubMed https://doi.org/10.2807/1560-7917.ES.2020.25.17.2000257

7. Robert C, Casella G. Monte Carlo statistical methods. New York: Springer Science and Business Media; 2013.

8. Wallinga J, Lipsitch M. How generation intervals shape the relationship between growth rates and reproductive numbers. Proc Biol Sci. 2007;274:599–604. PubMed https://doi.org/10.1098/rspb.2006.3754

Appendix Table 1. Estimated serial interval (SI) distributions*

	Median (95% credible interval)	
Type of infector–infectee pairs	Simulated SI	SD SI
All cases (N = 209)	3.28 (−5.41, 13.11)	4.62 (4.16, 5.25)
Cases with only 1 known contact (N = 93)	3.74 (−5.10, 13.91)	4.75 (4.11, 5.86)
Cases with multiple or no contacts (N = 116)	2.83 (−5.83, 12.83)	4.66 (4.05, 5.78)

*SI, Serial interval; DOO, Date of symptom onset.

Appendix Table 2. Subgroup analyses*

	Median (95% credible interval)			
Infectee type	Mean T_g	SD T_g	R_0	p
Cases with multiple known contacts (N = 72)	3.72 (2.57, 5.04)	3.22 (1.49, 5.43)	1.10 (1.07, 1.14)	0.69 (0.56, 0.81)
Cases in clusters with no missing DOOs (N = 103)	4.05 (3.13, 5.06)	2.90 (1.36, 4.42)	0.98 (0.97, 0.98)	0.64 (0.53, 0.74)

*T_g, generation time; SD, standard deviation; R_0, basic production number; p: pre-symptomatic proportion; DOO: Date of symptom onset.

Appendix Table 3. Sensitivity analyses of reproduction number R_0 distribution

				Median (95% credible interval)	
Infectee type	No. time points	Max daily incident cases	Growth rate of log (cases)	Original R_0	Revised R_0
Main analyses					
All cases (N = 209)	37	13	0.027	1.09 (1.08, 1.11)	1.90 (1.03 to 1.17)*
Cases with only 1 known contact (N = 93)	32	7	0.017	1.11 (1.08, 1.14)	1.07 (1.05, 1.09)
Cases with only multiple or no known contact (N = 116)	35	13	0.021	1.08 (1.06, 1.11)	1.06 (1.04, 1.08)
Subgroup analyses					
Cases with multiple known contacts (N = 72)	8	5	0.104	1.10 (1.07, 1.14)	1.39 (1.22, 1.57)
Cases in clusters with no missing DOOs (N = 103)	34	7	−0.003	0.98 (0.97, 0.98)	0.99 (0.99, 0.99)

*Estimates computed using resampled growth rates.

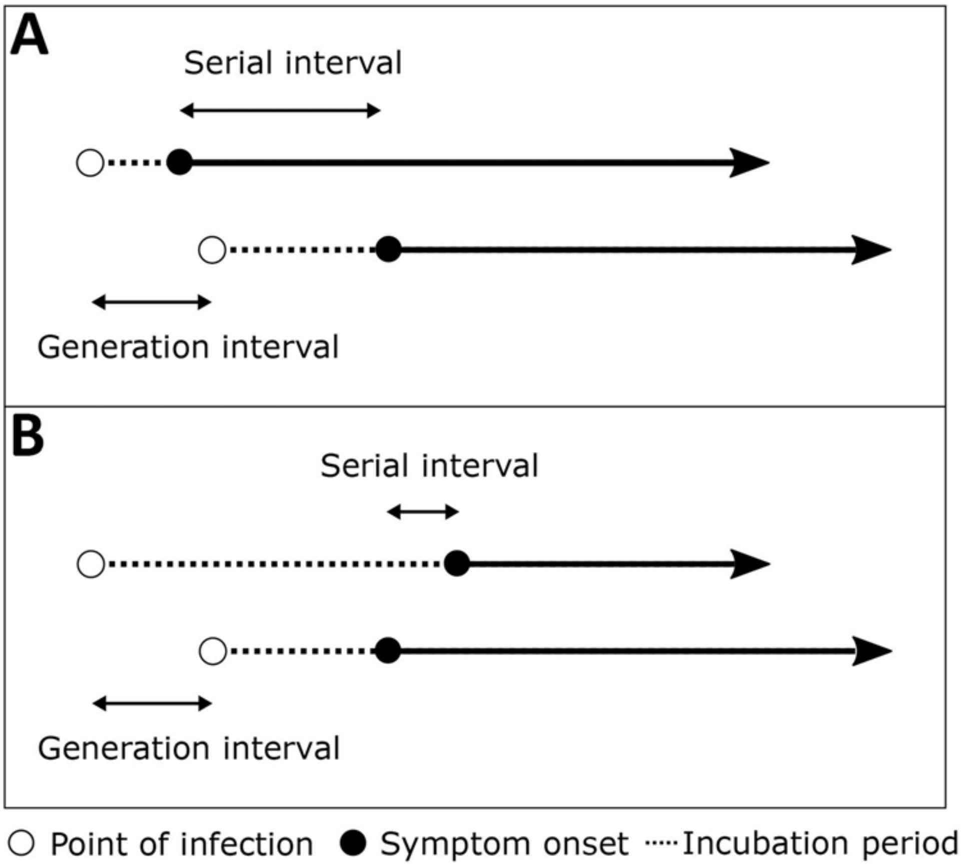

Appendix Figure 1. Time intervals within a transmission chain.

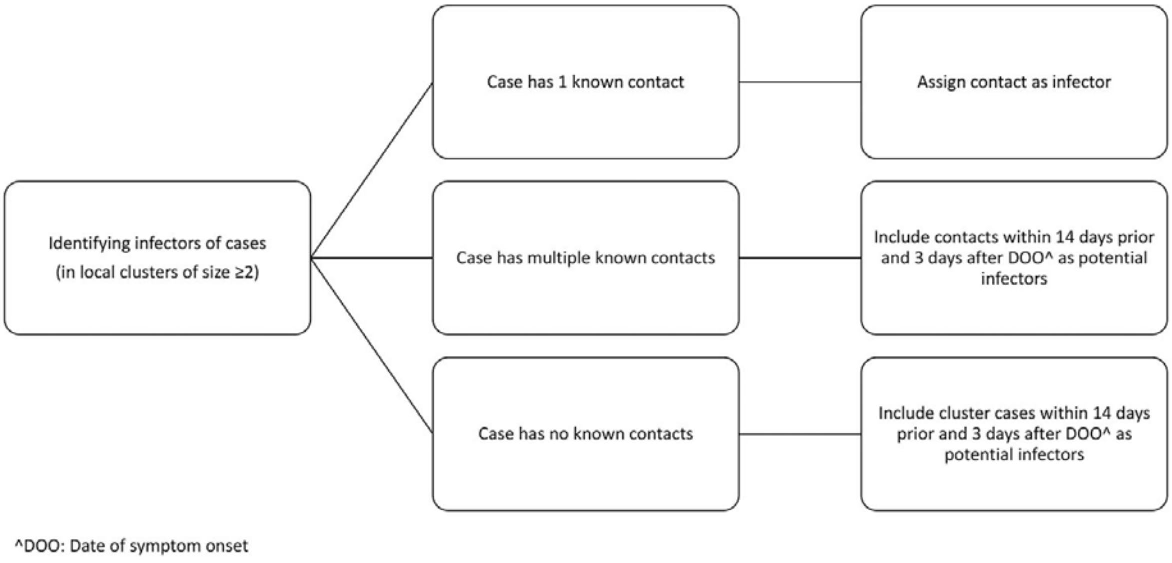

Appendix Figure 2. Heuristic for identifying potential infectors of a case.

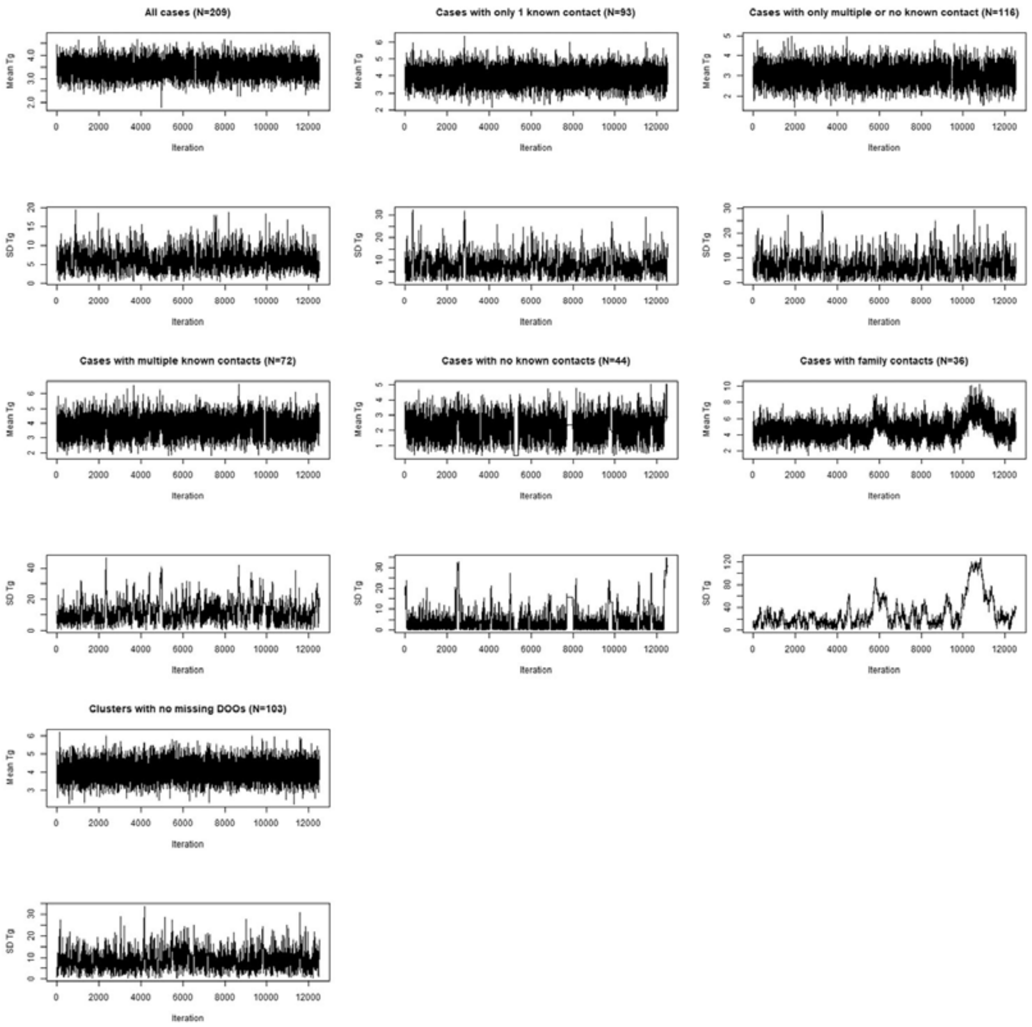

Appendix Figure 3. Convergence plots.
